# Packed-Bed Chemical Looping Reforming for Renewable Syngas Production from Glycerol with Ni_Fe-Based Oxygen Carriers/Catalysts

**DOI:** 10.1021/acs.energyfuels.4c02863

**Published:** 2024-10-16

**Authors:** Claudia Navarro, Adam Zaidi, Christopher de Leeuwe, Vincenzo Spallina, Gemma Grasa

**Affiliations:** † Environmental Research Group, 120031 Instituto de Carboquímica (Spanish National Research Council, ICB-CSIC), Miguel Luesma Castán 4, 50018 Zaragoza, Spain; ‡ Department of Chemical Engineering, 5292 University of Manchester, Manchester M13 9PL, U.K.

## Abstract

A chemical looping (CL) reactor can provide the energy required to produce syngas from the reforming of glycerol. This can be done through the dynamic operation of packed-bed (PB) reactors that consecutively cycle in a three-stage process that includes oxidation, reduction, and a reforming stage. In this work, Ni-/Fe-based materials were synthesized, characterized, and tested as oxygen carriers and glycerol reforming catalysts during chemical looping reforming in packed-bed (CLR-PB) reactors. Two materials were prepared via coprecipitation/impregnation with different Ni loads (4.3 and 12 wt %) and similar Fe loadings (9.9 and 8.5% wt) and later on granulated to produce particles in a size cut of 0.6–1 mm. The materials presented high catalytic activity in a wide range of reforming temperatures (650–800 °C), being able to achieve over 95% glycerol-to-gas molar conversion in very short gas/solid contact times and at relevant conditions suitable for process scale-up. The results indicated that it is possible to produce materials with a low Ni load, which present sufficient catalytic activity and thermochemical capacity thanks to the presence of Fe, to operate under the glycerol CLR-PB configuration.

## Graphical abstract

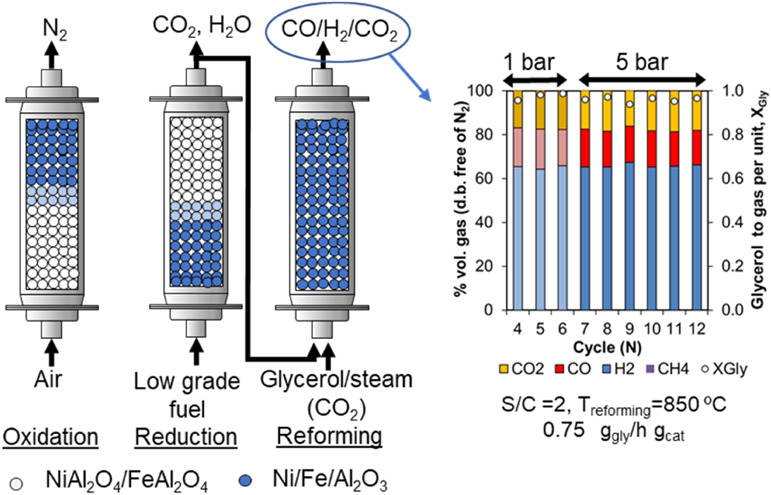

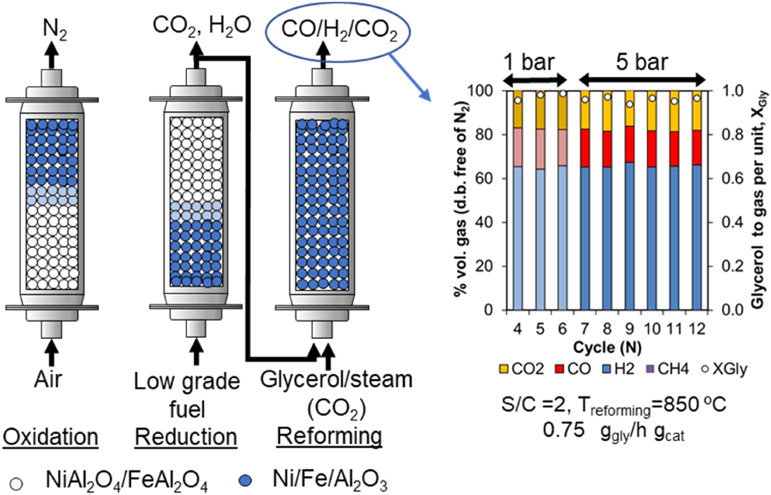

## 1 Introduction

The biodiesel industry has experienced important growth in the past decade emerging as an alternative to fossil fuels.^1^ The Renewable Energy Directive EC/2009/28 implemented by the EU, which mandates the use of 10% of renewable energy in transport in 2020 and an increment to 14% by 2030,^2^ will further contribute to accelerating biodiesel production. Glycerol (C_3_H_8_O_3_) constitutes approximately 10 wt % of biodiesel production byproducts. As a consequence, this has led to an excess supply of highly impure crude glycerol during the past decade, which is mainly incinerated,^3^ used for biogas generation,^1,4^ or even transferred to landfills. As an alternative, the integration of waste glycerol into renewable energy production processes has been proposed to increase the benefits of biodiesel production if properly treated.^5–7^ This work focused on the production of syngas from glycerol via chemical looping reforming (CLR) utilizing a synthesized Fe–Ni/Al_2_O_3_ oxygen carrier (OC). In recent years, CLR has appeared as an emerging technology, where CO_2_ separation is inherently integrated by using a solid metal oxide that acts as an OC.^8^ CLR processes have been proposed in different configurations, including fluidized bed systems operating at atmospheric pressure^8^ and dynamically operated packed bed reactors capable of operating at high pressure.^9–12^ High-pressure operation is preferable for both syngas uses in a downstream synthesis process and CO_2_ capture; therefore, chemical looping reforming in packed bed (CLR-PB) reactors have also been studied for a variety of processes, such as methanol production, ammonia production, or liquid fuel synthesis.^13,14^ This technology improves heat management,^15^ and it has been demonstrated in pseudocontinuous operation up to early TRL5 for CH_4_ steam reforming and CO_2_ reforming,^16^ showing promising economics for blue hydrogen production.^17^ Moreover, it is a flexible process that can be adequate for the reforming of diverse fuels, and it can be considered as carbon-negative when using crude glycerol as feedstock.^18,19^ The CLR-PB process has been designed to be operated in dynamic mode, in which the energy needed to sustain the highly endothermic glycerol reforming with steam (and/or CO_2_) is provided through the highly exothermic oxidation of an OC. Therefore, the CRL-PB process comprises three stages, as displayed in the simplified scheme shown in Figure 1. An initial stage can be considered as OC oxidation with air, which increases the temperature of the bed of solids, while a hot O_2_-depleted gas stream is obtained at the reactor exit. In the second stage, the OC is reduced with a low-grade fuel, such as waste from a downstream process (i.e., tail gas from Fischer–Tropsch). It generates a stream of CO_2_ and H_2_O, where CO_2_ can be easily separated at high purity by H_2_O condensation. This is a thermally neutral process stage, and the bed of solids is left in reduced form, hot, and active as a catalyst for the final glycerol reforming stage. By controlling the flow rates and cycle time during each process stage, it is possible to achieve a thermal neutral behavior in the reactor.^18^

**Figure 1:**
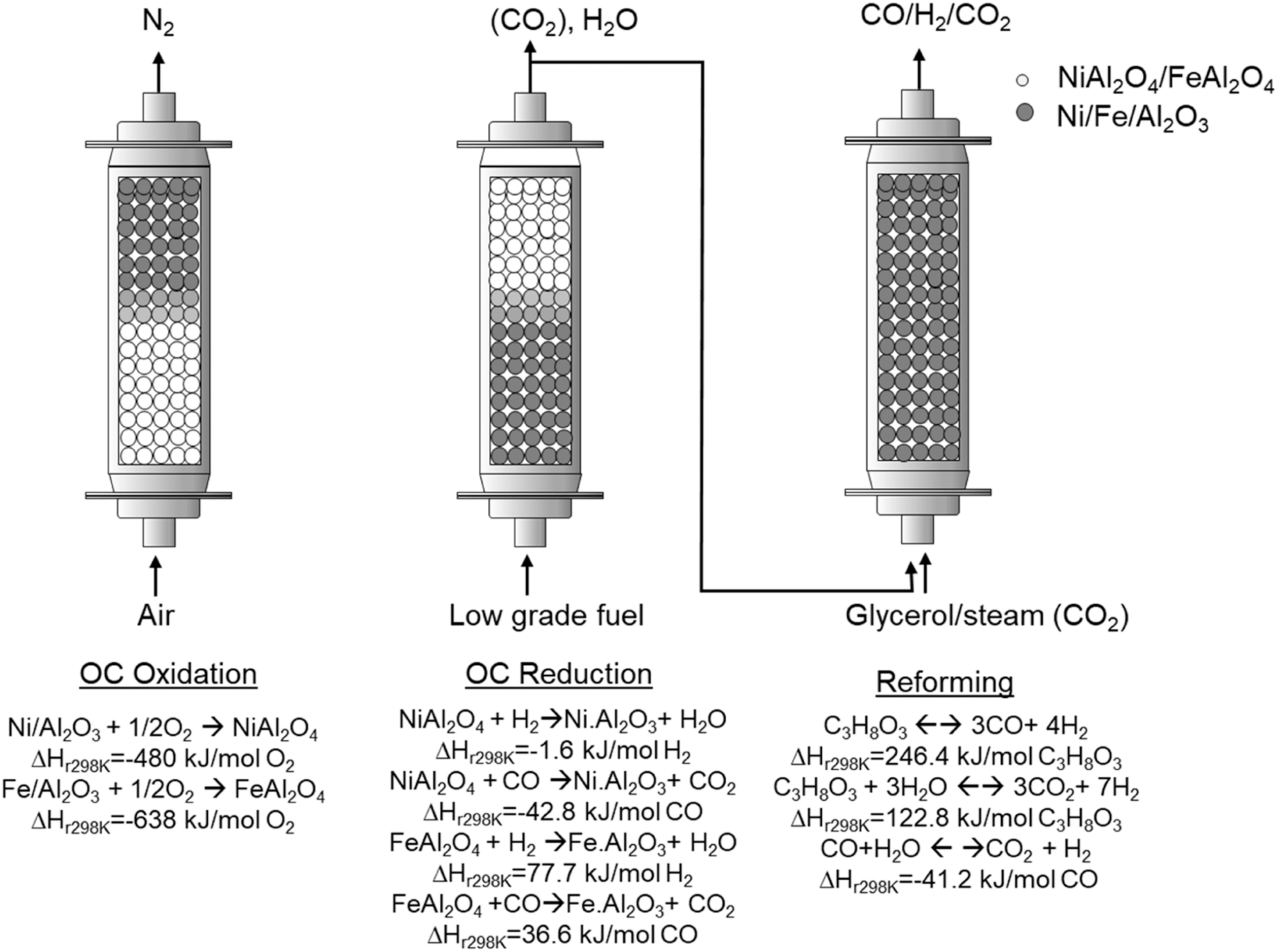
Three-stage glycerol chemical looping reforming in a packed bed (CLR-PB).

Ni-based materials have been mainly proposed in the literature as OCs/catalysts^8,20–22^ for CLR processes as they can sustain the energy balance for the whole process and present sufficient catalytic activity for reforming reactions in their reduced form.^11^ However, in recent years, either to reduce the presence of Ni or to improve OC/catalyst performance, alternative mixed metal oxides have been proposed. Among them, this work focused on the use of bimetallic Ni_Fe materials as Fe-based OCs have good reactivity,^23^ resistance to carbon build-up, and low toxicity.^24^ Moreover, their catalytic activity can be significantly increased when Ni is used to form a composite OC with Fe.^25^ In this way, Ni_Fe OCs have been used for Chemical Looping Combustion (CLC),^25^ in CH_4_ CLR processes,^26^ toluene or tar steam reforming,^27,28^ CH_4_ dry reforming,^29^ and recently, glycerol CLR.^30^ Focusing on CLR processes, perovskite-type OCs are among the most promising ones, as they present oxygen vacancies that can improve their redox properties,^30^ as well as materials susceptible to forming spinel-type structures.^31,32^ In this work, Al_2_O_3_ was proposed as a support to stabilize the active phases and to allow the use of materials with relatively low metal content.

The objective of this work is to prove at TRL3 (Technology Readiness Level 3: experimental proof of concept) the steam glycerol CLR-PB for syngas production along consecutive cycles with a Fe- and Ni-based spinel-type OC with low Ni content. Compared to existing studies in the field, this work presents for the first time the synthesis, characterization, and proof-of-concept of application on different reactor sizes of the bifunctional material and its optimum formulation with respect to glycerol-to-syngas conversion and gas–solid redox reactions. Therefore, the assessment includes material synthesis procedures, Ni/Fe content, and reactor operation variables (such as temperature, pressure, and glycerol weight hourly space velocity, WHSV) that are relevant for industrial applications.

## 2 Materials and Methods

### 2.1 Material Synthesis and Characterization

Two materials with distinctive Ni loads were prepared through the impregnation of a support consisting of iron and alumina. The synthesis was performed in two steps. First, iron supported on alumina was prepared via coprecipitation by mixing solutions with the appropriate concentration and proportion of Fe (1.5 M) and Al (0.3 M) nitrates and sodium carbonate as the precipitating agent (added dropwise in a 30% volume excess over stoichiometric value to ensure proper precipitation). After adjusting pH to 9 and 2 h aging at room temperature, the solution was filtered and washed to remove sodium traces. The material was then dried overnight (at 115 °C), ground (100–200 μm), and calcined in a furnace at 1000 °C for 4 h.^33^ The impregnation method consisted of mixing the support with a nickel nitrate solution and stirring at room temperature until the material turned into a slurry. The material was then dried (at 115 °C) and finally calcined at 875 °C for 2 h. In Table 1, the detailed compositions, as determined through inductively coupled plasma optical emission spectrophotometry, are reported. These compositions were chosen to test the performance of materials with distinctive Ni content. The material with the highest Ni content could resemble the Ni content of a conventional reforming catalyst, while the second material presents a much lower Ni content than conventional catalysts. The Fe content in the material was selected to allow for sufficient oxygen transport capacity (OTC) and to sustain the energy balance for the glycerol reforming stage. From the materials in powder form, particles with a particle size cut of 0.6–1 mm were produced via granulation in an EIRICH Laboratory Mixer Type EL1. A 20% weight PEG 6000 aqueous solution was chosen as a binder.

**Table 1:** Catalysts/OC Fe and Ni Contents

OC/catalyst	Fe (wt %)	Ni (wt %)	Fe/Ni molar ratio
Fe_10_Fe/Ni_2.4	9.9	4.3	2.4
Fe_10_Fe/Ni_0.8	8.3	12	0.8

The materials were characterized to determine the dispersion of metals, the crystalline species, and the dependence of their reducibility with temperature; their capability to transfer O_2_ along redox cycles and finally their textural properties were also assessed fresh and after use in the reactors along complete reforming/redox cycles. The dispersion of metals and their morphology were assessed by scanning electron microscopy (SEM) and energy-dispersive X-ray (EDX) scans. Samples were embedded in epoxy resin to take images at cross-cut. To determine the crystalline species, fresh and cycled materials were analyzed both oxidized and reduced via X-ray diffraction (XRD) using a Bruker D-8 Advance diffractometer with Cu Kα radiation. The ICDD-PDF2 database was used to identify phases. Catalyst performance as OC and its evolution along redox cycles were evaluated by thermogravimetric analysis (TGA), which had been described in detail in previous works.^33,34^ Up to 60 redox cycles were performed, where the continuous sample mass change was recorded during oxidation (20% O_2_ in N_2_) and reduction (20% H_2_/N_2_) at 900–920 °C. OTC was calculated according to eq 1:

$$\mathrm{OTC}=\frac{m_{O_{2}}}{m_{\mathrm{ox}}}=\frac{m_{\mathrm{ox}}-m_{\mathrm{red}}}{m_{\mathrm{ox}}}$$ 1

where *m*_ox_ is the mass of the fully oxidized material and *m*_red_ is the mass of the reduced sample. Finally, the material's textural properties (fresh and after use in the reactors) were determined through N_2_ physisorption at 77 K (ASAP 2020 Micromeritics) by applying the BET method to the obtained isotherm; solid density was determined through a He pycnometer (ACCUPYC II, Micromeritics); and porosity was determined through Hg porosimetry (AUTOPORE V, Micromeritics).

### 2.2 Experimental Setup

The materials were tested in three different PB reactors for different purposes. Material stability along redox cycles and under prolonged exposure at high temperatures was assessed at the University of Manchester (UoM) PB reactor, as shown in Figure 2a. The installation is equipped with the corresponding gas mass flow controllers; the core of the rig is a reactor made of a high-temperature-resistant stainless-steel tube (253MA) (30 mm internal diameter). Seven grams of Fe_10_Fe/Ni_0.8 material at a 0.6–1 mm particle size was loaded, resulting in approximately a 3 cm bed height. The inert material (quartz wool) at the bottom and top of the bed ensured that the material was held in the middle of the reactor and that the feed gases were preheated and homogeneously distributed along the cross section. The reactor was housed in a Carbolite furnace with temperature transducers at the reactor inlet and outlet. Experiments were conducted at atmospheric pressure and 5 bar_g_ in a temperature range of 500–900 °C. Outlet gas composition analysis was carried out using a HIDEN HPR-20 mass spectrometer. In this rig, the Fe–Ni material was tested for 100 h, during which it underwent 32 redox cycles.

**Figure 2:**
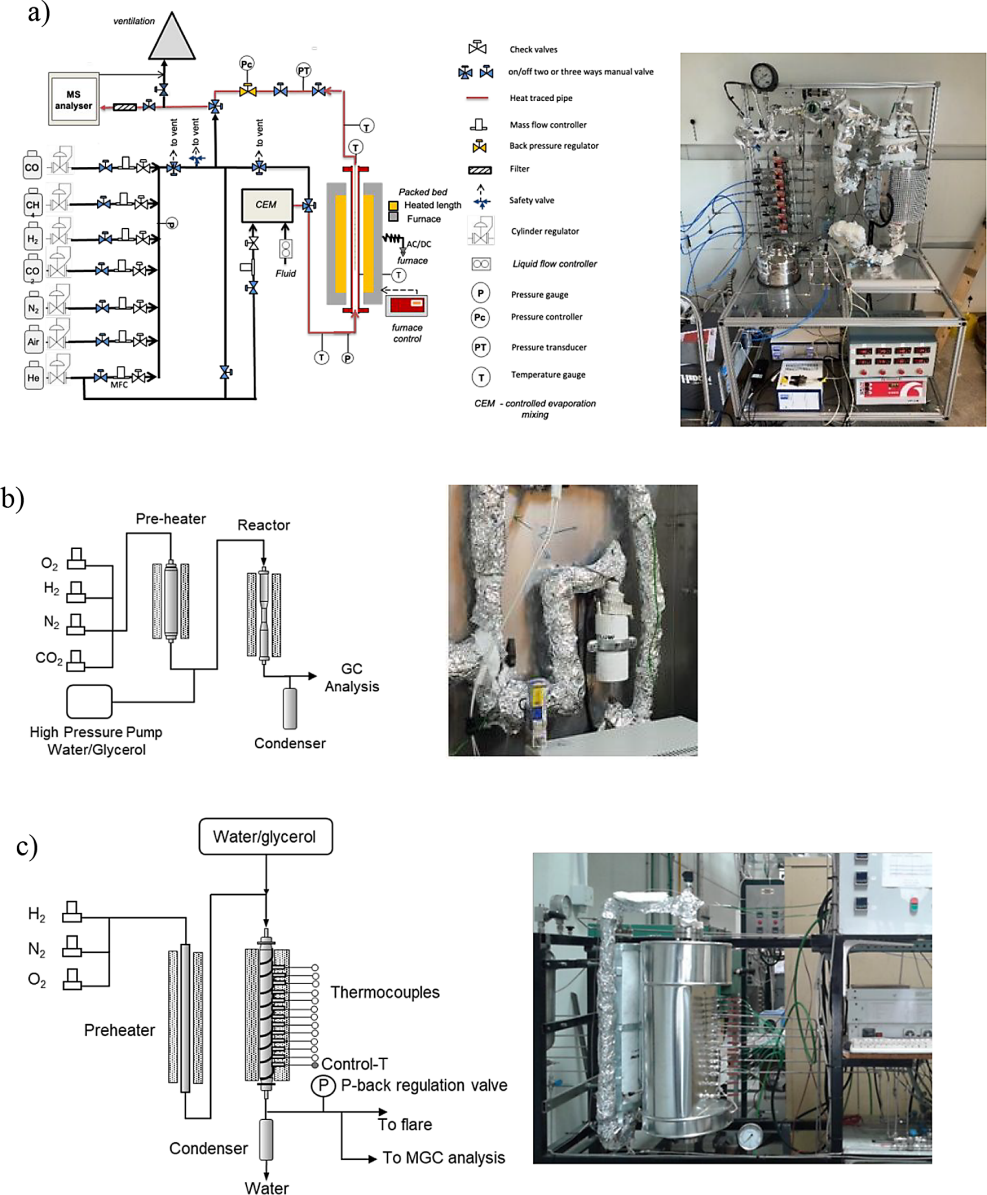
(a) UoM PB reactor for stability testing, (b) micro-PB reactor for isothermal reforming tests, and (c) TRL3 pseudoadiabatic PB reactor.

At the CSIC, the material performance as reforming catalyst and during complete consecutive glycerol reforming/oxidation/reduction cycles was assessed in two rigs. A quartz micro-PB reactor (4 mm internal diameter) placed inside an oven served to determine the catalyst performance under isothermal conditions in a temperature range of 650–800 °C (see Figure 2b). This reactor was capable of holding 200 mg of material in a particle size cut of 100–200 μm. Second, a TRL3 pseudoadiabatic PB reactor capable of operating under pressure was used to perform complete reforming/oxidation/reduction cycles (Figure 2c). The core of this installation is a PB reactor that consists of a stainless-steel tube with an internal diameter of 18 mm, which is externally heated by an electric heating wire (1.25 kW). The reactor is capable of holding up to 100 g of material. An external layer of insulation covers both the reactor and heating wire to reduce heat loss and to resemble adiabatic behavior. The control of temperature was carried out by a thermocouple immersed at the bottom part of the bed (the last portion of the bed facing the reaction). The axial temperature profile during the operation was measured by means of a series of thermocouples immersed along the reactor. The reacting gases (H_2_, O_2_, and N_2_) were fed into the reactor by using mass flow controllers, while water/glycerol mixtures (with the desired steam-to-carbon molar ratio, S/C) were fed with a high-pressure pump from the top part of the reactor through a trace-heated pipe and impulse with hot N_2_ that also served to close the mass balance to the system during reforming. At the reactor exit and downstream of the condenser vessel, a back-pressure regulator valve allowed the operation under pressure. Finally, gas analysis was performed by using a micro-GC (Varian CP-4900) apparatus. In this rig, the effects of glycerol spatial velocity, the operation under pressure, and the Ni content in the OC were assessed along consecutive cycles. During the reforming stages, the carbon balance was evaluated by the quantification of the carbon species in the gas phase and the total organic carbon determination analysis to the condensate recovered to determine the glycerol conversion and coke formation. The conditions of experiments carried out in the three apparatus are shown in Tables 2–4.

**Table 2:** Experimental Conditions during Redox Cycles at the UoM PB Reactor (Figure 2a)

	oxidation			reduction		
	*T*_bed_ (°C)	O_2_ (% vol)	*P* (bar_g_)	*T*_bed_ (°C)	H_2_ (% vol)	*P* (bar_g_)
Fe_10_Fe/Ni_0.8	500–700	10.5	1 and 5	700–900	20	1–5

**3:** Experimental Conditions during Glycerol Reforming under Isothermal Conditions at the Quartz Micro-PB Reactor (Figure 2b)

	reforming		
	*T*_bed_ (°C)	S/C	g gly/h g cat
Fe_10_Fe/Ni_0.8, Fe_10_Fe/Ni_2.4	650–800	2	3

**Table 4:** Experimental Conditions at the TRL3 Pseudoadiabatic PB Reactor along Consecutive Cycles (Figure 2c)

	oxidation		reduction		reforming		cycle
	O_2_ (% vol)	*P* (bar)	H_2_ (% vol)	*P* (bar)	g gly/h g_cat_	*P* (bar)	
Fe_10_Fe/Ni_0.8	10–21	1	35	1	0.3–0.7	1	1–6
	21	1–5	35	1–5	0.7	2.5–5	7–12
Fe_10_Fe/Ni_2.4	21	1	35	1	0.5	1	1
	21	5	35	5	0.5	5	2–6

The initial bed temperature (*T*_bed_) during oxidation was set at 620 °C (and 720 °C for the material with lower Ni content), while during reduction and reforming, the initial *T*_bed_ was set at 850 °C. A steam-to-carbon, S/C, molar ratio was equal to 2; hence, the H_2_O-to-C_3_H_8_O_3_ molar ratio was equal to 6. Maximun operation pressure was determined by reactor specifications. Operating the process under pressure would benefit the CLR-PB process, as the global objective for CLR-PB is to integrate the syngas produced in a downstream process for Fischer–Tropsch fuel synthesis.

## 3 Results and Discussion

### 3.1 Material Characterization

Material characterization in powder form was extensively reported in a recent paper that focused on analyzing its reducibility in a TGA apparatus under high-pressure conditions.^33^ To summarize the results obtained from XRD characterization in powder form and reported by Palone et al.,^33^ it can be said that the analysis of the coprecipitated/impregnated materials (fresh, oxidized materials) revealed the presence of FeAl_2_O_4_ and NiAl_2_O_4_ spinels in addition to Al_2_O_3_. The crystalline species detected in the reduced samples varied, depending on the reduction temperature. When reduction was performed at 900 °C, Fe and Ni aluminates were reduced to a Fe–Ni alloy. The peaks corresponding to this Fe–Ni alloy appeared with an angle that was slightly shifted depending on the Fe/Ni ratio, in line with the results reported in the literature.^27,28,35^ At lower reduction temperatures (between 900 and 800 °C), the presence of Ni and Fe aluminates together with the Fe–Ni alloy was detected, and the intensity of the reflection peak for the aluminates was inversely proportional to temperature (i.e., lower reduction temperatures presented a more intense reflection peak for the aluminates).^33^ XRD analysis of OC/catalyst particles (fresh oxidized and after being used in the TRL3 reactor in reduced form) performed in this work revealed the same crystalline species as for the powdered materials (see Figure 3a,b). The fresh OC/catalysts, in the oxidized form, presented Al_2_O_3_ (PDF 42-1468), Fe, and Ni spinels in their structure (FeAl_2_O_4_ PDF 07-0068, NiAl_2_O_4_ PDF 81-0718). NiO (PDF 47-1049) was also detected in the material with the highest Ni content (Fe_10_Fe/Ni_0.8; see Figure 3a). The analysis of the cycled materials, in reduced form after their use in the reactor, revealed the presence of the Ni–Fe alloy (PDF 65-3244), together with Ni and Fe aluminates and Al_2_O_3_. The presence of aluminates in the reduced samples extracted from the reactor (reduced at an average T of 850 °C) was coherent with the characterization presented by Palone et al.^33^

**Figure 3:**
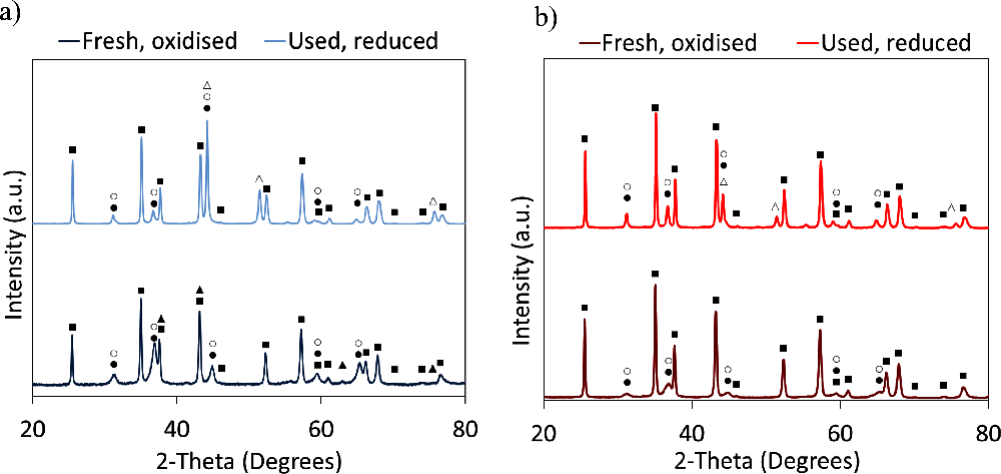
XRD patterns of (a) Fe_10_Fe/Ni_0.8 and (b) Fe_10_Fe/Ni_2.4. Samples were analyzed fresh (oxidized) and after being cycled in the TRL3 reactor (reduced). Phases are denoted as Al_2_O_3_ (■), FeAl_2_O_4_ (●), NiAl_2_O_4_ (○), NiO (▲), and Fe–Ni alloy (△).

From the TGA analysis, it was determined that both materials presented high stability along redox cycles (see conditions in Section 2). As expected, the materials' OTC was dependent on their Ni (and Fe) content; therefore, Fe_10_Fe/Ni_0.8 was capable of transporting 0.059 g O/g OC, while Fe_10_Fe/Ni_2.4 presented an OTC of 0.028 g O/g OC, as both represented in Figure 4. Both materials improved their ability to transport O_2_ during the initial cycles, and then, they reached their maximum OTC according to their composition (assuming reduction to metallic Ni and Fe) that remained stable for the 60 cycles performed.

**Figure 4:**
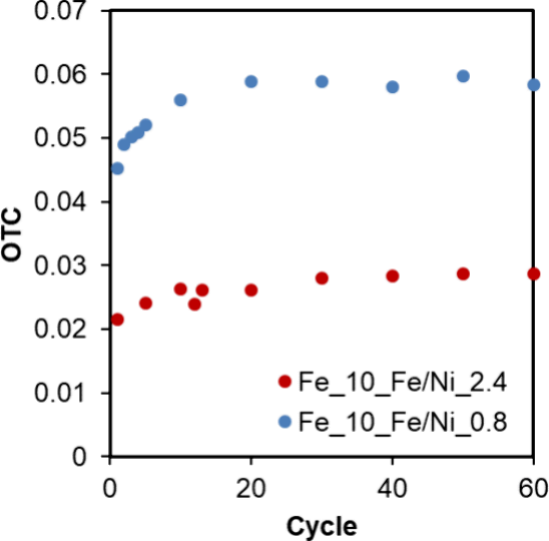
Materials' OTC with the number of oxidation/reduction cycles. Oxidation with 20% vol. O_2_ in N_2_ and reduction with 20% H_2_ in N_2_, both stages at 900 °C.

SEM-EDX analyses performed on the fresh oxidized and cycled materials in reduced form from the TRL3 reactor also corroborated the material stability along redox cycles. Figure 5 (right) shows an example of Ni and Fe distribution across the diameter of one fresh (oxidized, upper row) and one cycled particle (in reduced form, lower row) for each material's cross-sectional diameter ((a) Fe_10_Fe/Ni_2.4 and (b) Fe_10_Fe/Ni_0.8). The figure shows how the homogeneous distribution of Ni and Fe along the particle cross section was maintained after being exposed to multiple reforming/redox cycles. No apparent signs of Ni or Fe migration to the external layer of particles were detected.

**Figure 5:**
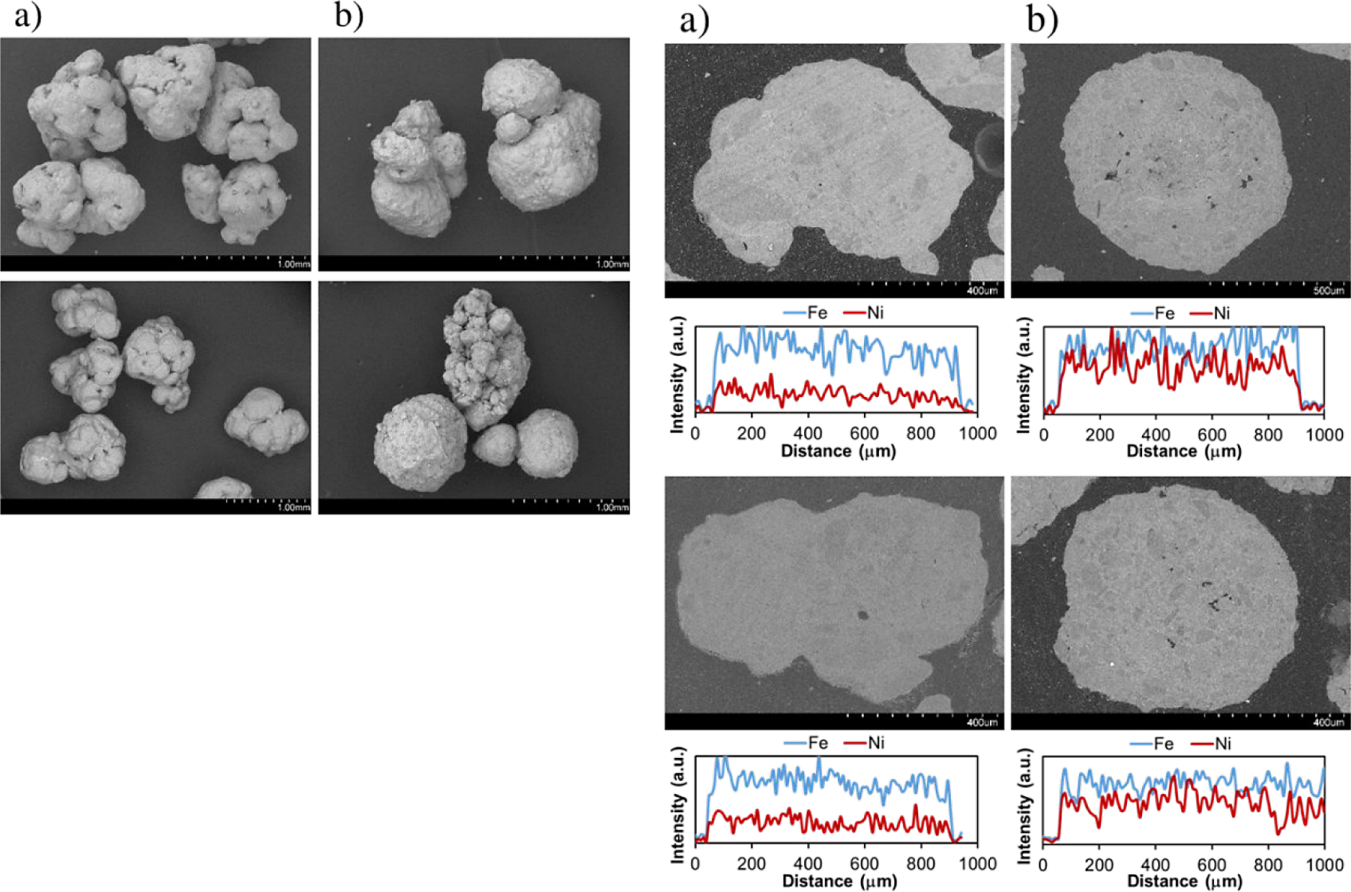
(Left) SEM images of particles and (right) SEM-EDX line scan of cross-cut particles for materials (a) Fe_10_Fe/Ni_2.4 and (b) Fe_10_Fe/Ni_0.8. Upper row: fresh, lower row: cycled material after use in the TRL3 reactor.

Finally, Table 5 contains the material’s textural characterization fresh and after their use in the TRL3 reactor. A representative sample of each material was taken and sent for analysis, as described in Section 2.1 As can be observed, both materials presented a porosity in the range of 45–48% that was maintained after being tested in the TRL3 reactor (approximately 50 h under process conditions described in Table 4, which included the operation of the reactor at temperatures between 600 and 875 °C and pressures of 1 and 5 bar exposing the materials to temperature variations due to the exothermic or endothermic nature of the reactions). Materials' characterization after their use in the reactor showed that the BET surface area for the material with higher Ni content was reduced compared to the material with the lowest Ni content, whose BET surface area was barely affected by operation in the reactor. The OTC variations observed in the initial redox cycles can be related to the stabilization of material's crystalline structure, which might also be reflected in the decrease of BET surface area observed in the Fe_10_Fe/Ni_0.8 OC/catalyst. No signs of a decrease in catalytic activity or redox properties have been observed during the operation in the reactor that could be caused by this decrease in BET surface area.

**Table 5:** Materials' Textural Characterization Fresh (Oxidized) and After Use in the TRL3 Pseudoadiabatic PB Reactor (in Reduced Form)

OC/catalyst	fresh (oxidized)			cycled (reduced)		
	ε (%)	ρ (g/cm^3^)	*S*_BET_ (m^2^/g)	ε (%)	ρ (g/cm^3^)	*S*_BET_ (m^2^/g)
Fe_10_Fe/Ni_2.4	44.8	4.13	8.9	44.9	4.15	7.0
Fe_10_Fe/Ni_0.8	48.37	4.17	14.0	47.63	4.27	7.8

### 3.2 Material Performance along Oxidation and Reduction Cycles in a Fixed Bed Reactor

To analyze in detail the behavior of the OC/catalysts along redox cycles in a PB reactor that can be operated under pressure, the material Fe_10_Ni_Fe/Ni_0.8 was tested at the UoM installation (Figure 2a). Upon initial use, the material was stabilized by performing cycles at 700 °C that alternated reduction stages using 10% vol H_2_ and oxidation stages with 10.5% vol O_2_. To assess the material performance during oxidation, consecutive cycles were performed at different temperatures (500–700 °C) and pressures (1–5 bar_g_). After oxidation, the material was reduced at a constant temperature of 900 °C in 20% H_2_ in N_2_, as recommended by TGA testing on the material.^33^ Figure 6a shows an example of the results obtained during oxidation at 5 bar_g_. It can be observed in Figure 6 that increasing the oxidation temperature slightly increased the level of the O_2_ uptake, indicating that there was a certain dependence of the OTC with temperature. Regarding the breakthrough slope, it was similar in this range of temperatures (except for the test at the lowest temperature), indicating that the kinetics were similar at these pressure and temperature ranges (Figure 6a). From the O_2_ breakthrough, the material’s OTC was calculated as a function of oxidation temperature (presented in Figure 6b) and referred to the material's maximum OTC according to the composition. Figure 6a shows that material oxidation was slightly affected below 600 °C; however, it was still capable of transporting 90% of its maximum OTC at 500 °C for both experiments at 1 and 5 bar_g_ (Figure 6b).

**Figure 6:**
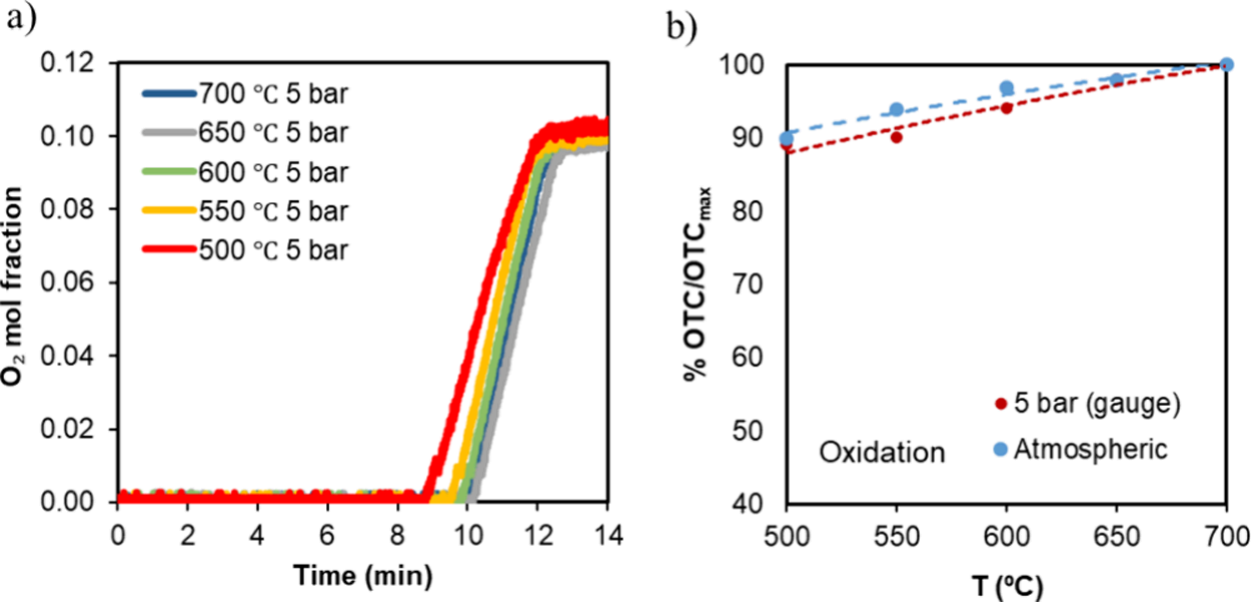
(a) O_2_ breakthrough at the reactor exit at 5 bar_g_. (b) Material's OTC with temperature, as determined from oxidation and referred to its maximum OTC according to the composition. Oxidation performed with 10.5% vol O_2_ in N_2_. In every case, after oxidation, reduction was performed at 900 °C with 20 vol % H_2_ in N_2_.

To assess the material performance along reduction, the temperature was varied in the range of 700–900 °C with 50 °C intervals at atmospheric pressure and 5 bar_g_. During the reduction experiments, the oxidation was kept constant at 700 °C (in a 10.5% vol O_2_). Figure 7a shows an example of experiments at atmospheric pressure, and it can be seen how the breakthrough of H_2_ is affected by temperature. The figure also includes two experiments performed at 5 bar_g_. In all the temperatures, there is an initial fraction of time, where no H_2_ is measured at the reactor outlet, indicating that H_2_ was completely consumed by the OC reduction. From that point, the slope of the breakthrough curve was affected by temperature, demonstrating the dependence of kinetics on temperature that had been previously observed through TGA and XRD.^33^ Operating the system at 5 bar_g_ helped to limit the effect that reduction *T* had on the material's OTC, but its reduction reaction rate still presented a strong dependence on temperature (see Figure 7b).

**Figure 7:**
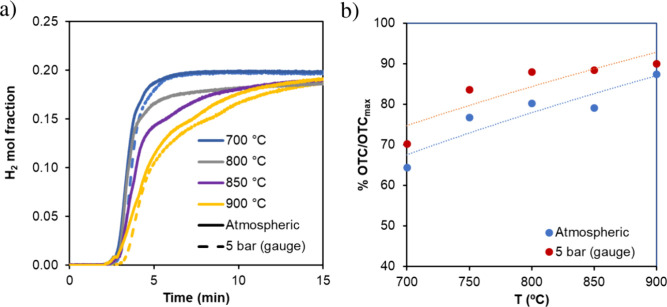
(a) H_2_ breakthrough at the reactor exit at atmospheric pressure (and 5 bar_g_ for temperatures of 700 and 900 °C). (b) Material's OTC with temperature, as determined from reduction and referred to its maximum OTC according to the composition. Reduction was performed with 20 vol % H_2_ in N_2_. In every case, after reduction, oxidation was performed at 700 °C with 10.5 O_2_ % vol O_2_ in N_2_.

### 3.3 Catalyst Performance on Glycerol Reforming

#### 3.3.1 Isothermal Conditions

The performance of the materials as glycerol reforming catalysts was assessed in the micro-PB reactor, as shown in Figure 2b, for a fixed S/C molar ratio of 2, a fixed glycerol spatial velocity (3 g_gly_/h g_cat_), and as a function of reforming temperature. From these experiments, product gas composition, glycerol-to-syngas conversion, and coke deposition were determined. These conditions resulted in gas velocities in the reactor between 0.174 and 0.202 m/s and gas–solid contact times between 0.094 and 0.075 s. Figure 8 shows an example of the results obtained, which corresponded to 2 h of stable operation under reforming conditions, with a product gas composition variation of ±0.2 vol %. Both materials resulted in glycerol molar conversion to gas higher than 95% for reforming temperatures of 700 °C or higher, while 93% conversion to gas was obtained in the test performed at 650 °C (with the catalyst with the highest Ni content). Product gas composition was close to thermodynamic predictions, and no side products were detected, except for the test performed with the material with 4.3% wt Ni at 700 °C. In this case, CH_4_ content was slightly higher than expected (0.71% vol vs 0.17% vol). The ratio of H_2_/CO decreased with increasing temperature, as expected according to the thermodynamic equilibrium for the WGS reaction (see the reactions occurring in Figure 1). Coke formation increased with decreasing reforming temperature for the material with higher Ni content (Figure 8a), in agreement with trends found in the literature,^36^ indicating that the activity of the catalyst is affected by reforming temperature, especially at temperatures below 700 °C. The material with 4.3% wt Ni presented a slightly higher coke formation rate, which was less dependent on temperature (Figure 8b); this indicates that this material presents lower catalytic activity and highlights the relevant role of Ni as an active phase in reforming reactions. The materials presented in this work were able to convert to syngas 3 g_gly_/h g_cat_ (47,000 h^–1^ refers to all the gas entering the reactor per catalyst volume) at a relatively low reforming temperature with low carbon deposition. This represents a clear improvement with respect to Ni-based CLR OC/catalysts^37^ or bimetallic catalysts/OC found in the literature.^5,36^ It can be concluded from this analysis that both materials presented sufficient activity as glycerol reforming catalysts in a range of temperatures that allowed for reactor dynamic operation in adiabatic systems.

**Figure 8:**
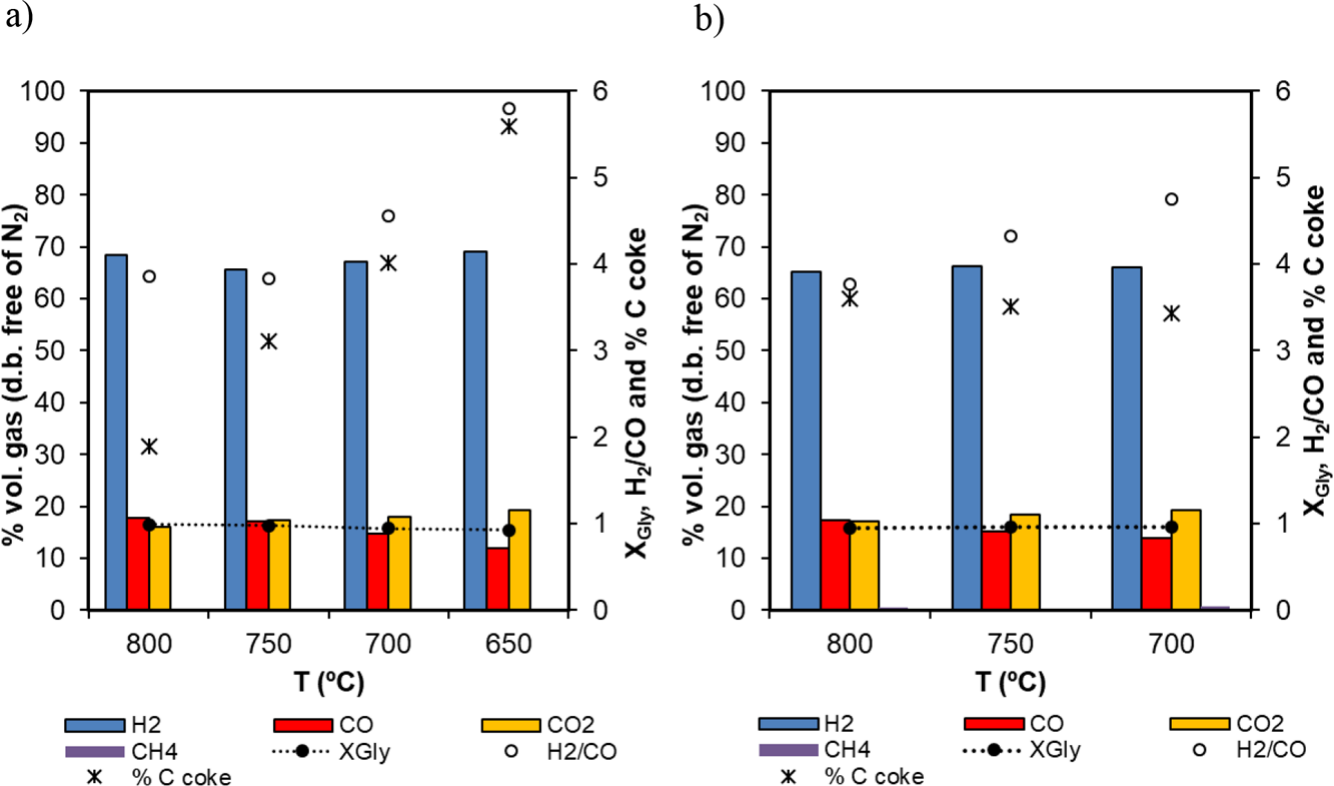
Catalyst performance as a function of reforming temperature. (a) Fe_10_Fe/Ni_0.8 and (b) Fe_10_Fe/Ni_2.4. The figures include product gas composition on dry basis, glycerol conversion to gas (*X*_gly_ per unit), H_2_/CO molar ratio, and the % of C moles forming coke. S/C molar ratio = 2, and WHSV = 3 g_gly_/h g_cat_.

#### 3.3.2 Complete Cycles

Finally, consecutive glycerol reforming/oxidation/reduction cycles were performed at the TRL3 scale in the pseudoadiabatic PB reactor, as shown in Figure 2c. In this reactor, it was possible to observe the evolution of temperature profiles with time and bed length, producing relevant information for the scaling-up of CLR in a fixed bed. The CLR process described in Section 1 operates in dynamic mode. This means that heat generated during the oxidation stage and stored in the bed of solids provides the required energy for the endothermic glycerol reforming reaction and subsequent adiabatic cooling of the bed. Figure 9 shows an example of results obtained during a complete reaction cycle starting from oxidation, reduction, and finally, followed by the glycerol steam reforming stage (cycle 4 in Table 4, material Fe_10_Fe/Ni_0.8). This cycle was performed at atmospheric pressure, and the bed was oxidized with air at an inlet gas velocity of 0.17 m/s; the reduction was performed in a 35% vol H_2_ in N_2_ at an inlet gas velocity of 0.21 m/s, and finally, the glycerol reforming stage was performed at 0.7 g_gly_/h g_cat_ with an S/C equal to 2 and 0.45 m/s inlet gas velocity. The upper line in the figure represents the gas composition obtained at the reactor outlet (on a dry basis) with time, while the lower row of figures shows the evolution of the temperature profile with time and bed length (in the legend). In this example, it can be observed how the progress of the reaction front could be followed through the temperature profile evolution in the bed. This was more evident during the exothermic oxidation stage. As an example, within the OC oxidation, it can be observed that during the pre-breakthrough, all the oxygen introduced in the reactor was consumed to oxidize the material, releasing heat that increased bed temperature slice by slice. When the reaction front reached the last thermocouple in the bed (*T*_c_ at bed length *L* = 12.5 cm), O_2_ started to break through the bed, and it was detected in outlet gas. As the reactor was not completely adiabatic due to heat loss, the temperature measured on each thermocouple as the oxidation front advanced deviated from the heat plateau predicted by oxidation models in the ideal adiabatic case.^11^ ^,^ ^38^

**Figure 9:**
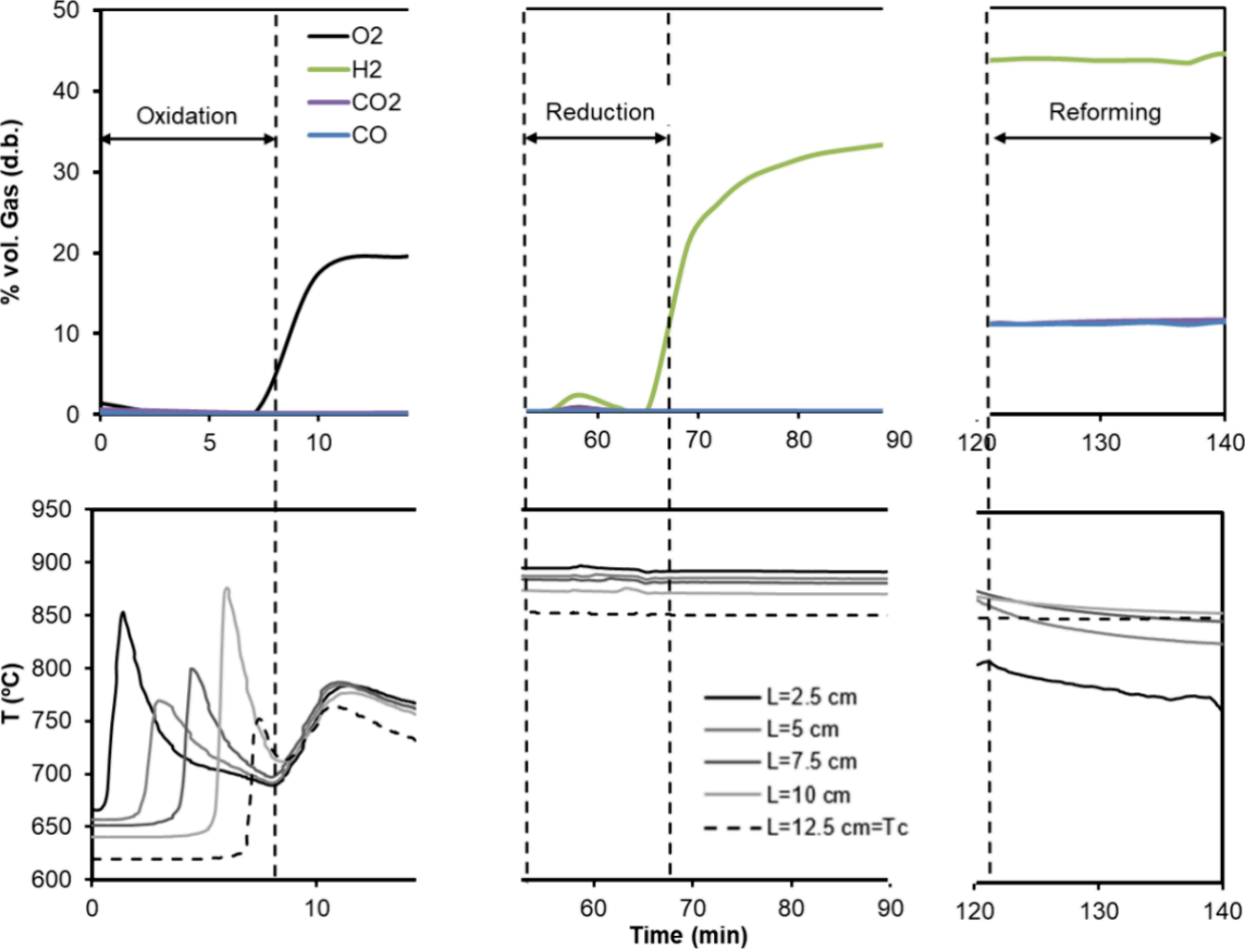
Upper row: example of gas compostion at the reactor outlet for a test performed with the Fe_10_Fe/Ni_0.8 material at 1 bar (Fe_10_Fe/Ni_0.8 cycle 4, in Table 4). Oxidation, reduction, and reforming stages are represented. Lower row: temperature profile evolution with time and bed length (see the legend) along the same test.

Focusing on the glycerol reforming stage, it was highly endothermic (see Figure 1 for enthalpies), and as observed in Figure 9 (lower row, right side), the bed solid temperature decreased progressively with bed length and time. However, the product gas composition was not affected by this progressive cooling as the gas left the reactor at equilibrium with the hottest temperature measured in bed. This might be due to the fact that the syngas produced in the reforming front reacted according to the WGS equilibrium along the bed length that was at a higher temperature.

To determine the effect that glycerol spatial velocity had on the OC/performance, a water/glycerol mixture (45 wt % glycerol, resulting in an S/C molar ratio of 2) preheated at 850 °C was used as the feed stream. The volumetric flow ranged from 0.75 to 1.2 mL liquid/min, resulting in gas velocities at the reactor inlet between 0.35 and 0.41 m/s, and the glycerol spatial velocities referred to the catalyst were 0.4, 0.6, and 0.7 g of gly/h g of cat (with gas/solid contact time ranging from 0.35 to 0.41 s). The bed of solids contained a total amount of 65 g of solids (52 g of Fe_10_Fe/Ni_0.8 mixed with 13 g of inert material, silicon carbide SiC) that resulted in a bed length of 15 cm. Figure 10 shows how the main effect of increasing the water/glycerol mixture WHSV introduced in the reactor was observed on the evolution of temperature profile with time and bed length, and this was due to the increased energy demand from the reforming reactions when they occurred at higher WHSV. As can be seen in Figure 10a, a product gas containing 65.6% H_2_, 17.42% CO, and 17.47% CO_2_ with negligible CH_4_ was obtained at the reactor exit (regardless of glycerol spatial velocity), resulting in a glycerol-to-syngas conversion of 97% and a H_2_/CO molar ratio of 3.79.

**Figure 10:**
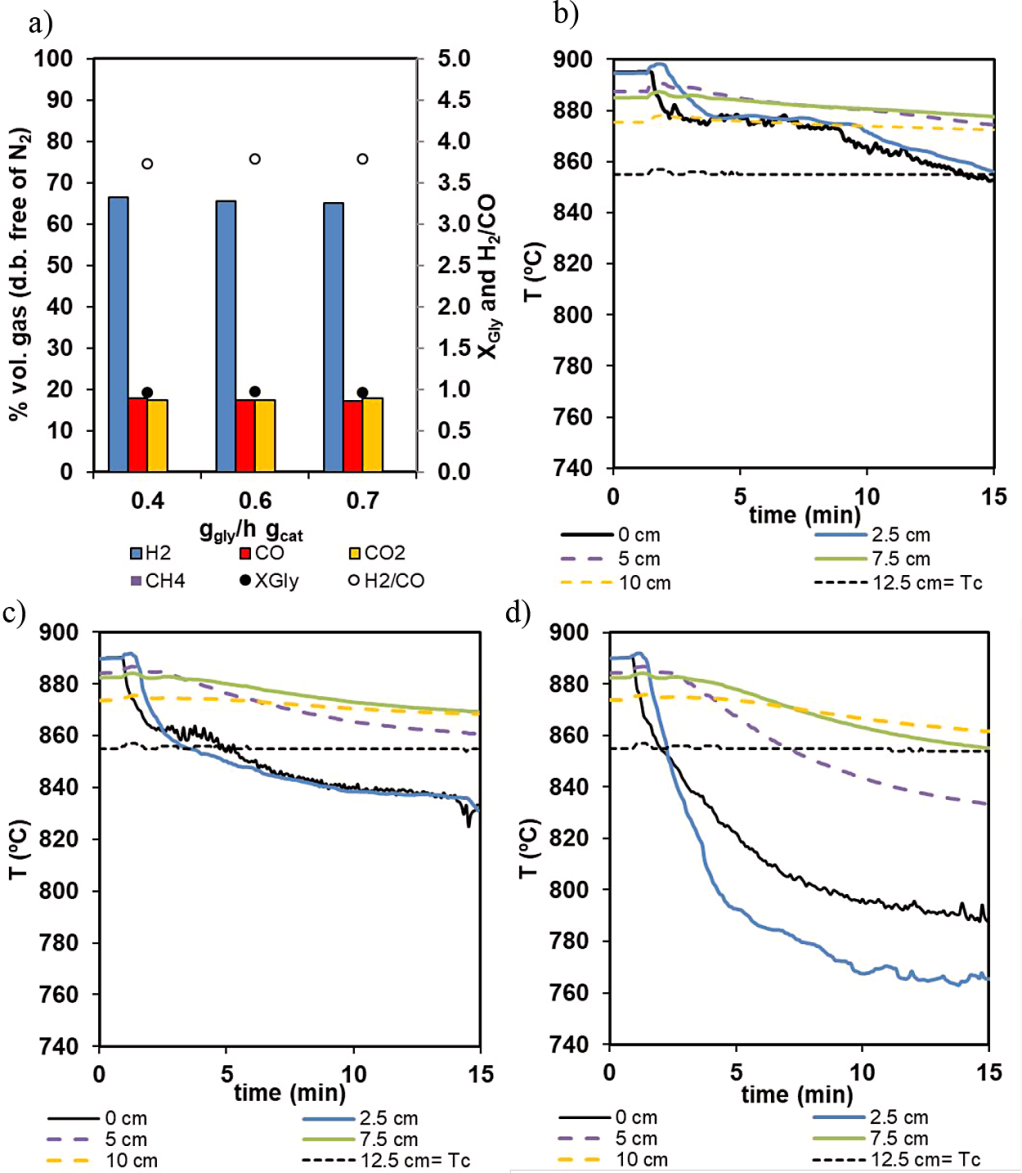
(a) Product gas composition as a function of glycerol WHSV (material Fe_10_Fe/Ni_0.8, cycles 1–3 in Table 4). (b–d) Temperature profile evolution with bed length and time for 0.4, 0.6, and 0.7 g gly/h g cat, respectively.

These results were in line with those obtained in the micro-PB reactor operating under isothermal conditions, as shown in Figure 8, where the catalyst operating at an even higher WHSV achieved almost complete glycerol conversion to gas. In Figure 10b–d, it can be observed that the main effect of increasing the glycerol/water mixture flow was that the energy demand increased to sustain the reforming reaction at increased WHSV and, as a consequence, a faster adiabatic cooling was observed. Figure 10b–d illustrates the evolution of the temperature profile over the bed length. As can be observed, there was a drop in the temperature measured in the bed. At the lowest spatial velocities tested, from the temperature profiles, it can be extracted that the endothermic reactions mainly take place in a very narrow bed length causing an evident cooling in this zone. As the glycerol/water feeding rate increased, a more drastic drop in temperature in the initial part of the bed and also a cooling trend in a wider bed length were observed. This indicates that the glycerol reforming reaction occurred in a wider bed length, and the product gas composition evolved to leave the bed at equilibrium with *T* of the last portion of the bed of solids. A Δ*T* of approximately 200 °C was measured for the highest spatial velocity. To assess the stable performance of the material, Fe_10_Fe/Ni_0.8 consecutive oxidation/reducing/reforming cycles at atmospheric pressure (cycles 4–6) and at 5 bar_g_ pressure (cycles 7–12) were carried out.

Figure 11 summarizes the results obtained from the consecutive reforming stages. As can be observed, very consistent results were obtained in terms of product gas composition during reforming, with glycerol conversion to gas (*X*_gly_) in the range of 0.96–0.99 at atmospheric pressure, and from 0.95 to 0.97 at 5 bar_g_, with low coke deposition (below 1 and 2% at 1 and 5 bar, respectively) and negligible CH_4_ selectivity. Catalyst activity along the cycles has not been affected by this low coke deposition, as the reforming stage in one cycle is followed by the oxidation stage from the following cycle, where coke will be oxidized, cleaning the catalyst surface.

**Figure 11:**
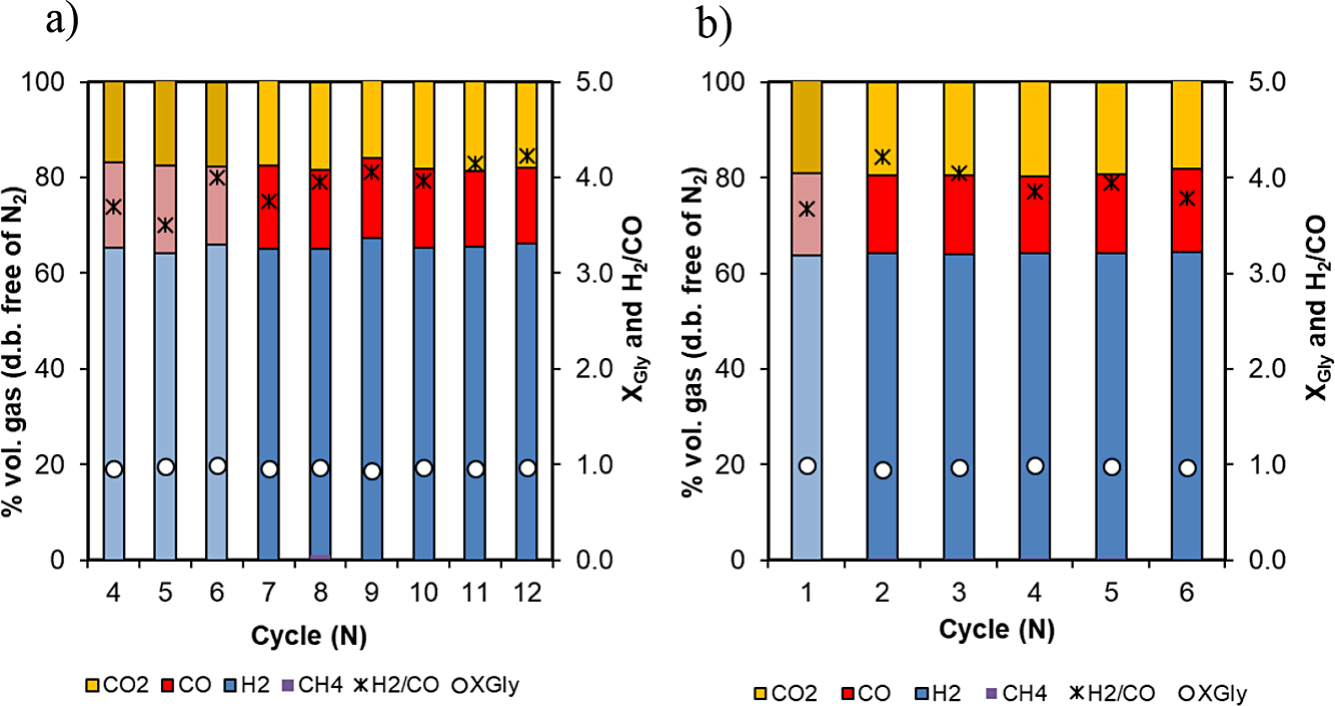
Product gas composition during the reforming stage along multiple cycles of reforming/oxidation/reduction: (a) Fe_10_Fe/Ni_0.8 and (b) Fe_10_Fe/Ni_2.4. Detailed operational conditions are given in Table 4. This figure includes glycerol conversion to gas, *X*_gly_, and the ratio of H_2_/CO obtained on the secondary axis.

Also, from these consecutive cycles, it was extracted that highly reproducible oxidation and reducing stages were also observed in terms of pre-breakthrough duration and maximum temperatures achieved during oxidation, as reported in Figure 12, for cycles 10 and 12 from Table 4. In this example, oxidation was performed with air at 5 bar_g_ and an inlet gas velocity of 0.04 m/s.

**Figure 12:**
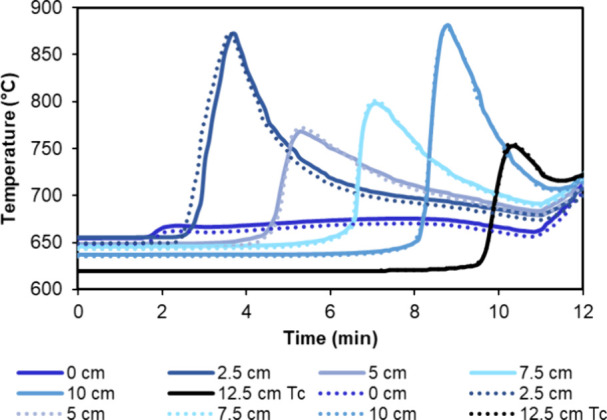
Temperature profile evolution with time and bed length during the oxidation stage for cycles 10 and 12 performed with the material Fe_10_Fe/Ni_0.8. Dashed lines correspond to cycle 10, and continuous lines correspond to cycle 12.

A similar set of experiments was performed with the material Fe_10_Fe/Ni_2.4, which contains only a 4% wt Ni (see Table 4 for detailed conditions of consecutive cycles). The main effect observed due to different Ni loads in the OC/catalyst was observed on the maximum Δ*T* measured during the oxidation stage (see Figure 13) and on the duration of the pre-breakthrough during oxidation and reduction stages, while no relevant differences were observed on product gas composition during reforming or in glycerol-to-syngas conversion (see Figure 11b).

**Figure 13:**
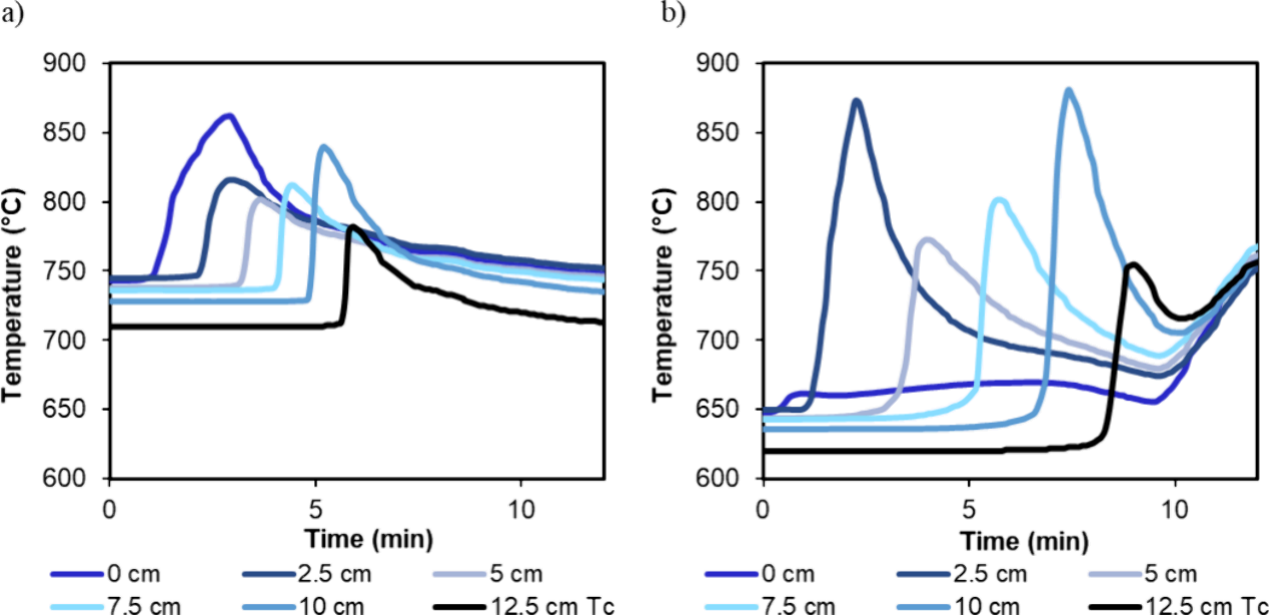
Temperature profile evolution with time and bed length during an oxidation stage: (a) Fe_10_Fe/Ni_2.4, cycle 5 and (b) Fe_10_Fe/Ni_0.8 cycle 10.

The results indicate that by adjusting the Ni and Fe load in the OC/catalyst, it is possible to design a material that could provide a thermally balanced process of the CLR-PB of glycerol and, at the same time, limit the Ni content. As an example, a material with 12% Ni (and 8.3% wt Fe) could increase the bed temperature up to 250 °C and compensate for the endothermic reforming stage operating at 0.7 g_gly_/h g_cat_. When the Ni content is lowered to 4 wt % (and Fe 9.9 wt %), a maximum Δ*T* of 140 °C has been measured, with a total amount of heat generated to compensate for the reforming stage operating at 0.5 g_gly_/h g_cat_.

## 4 Conclusions

Ni- and Fe-based OC/catalyst samples were synthesized through a coprecipitation/impregnation route with alumina as a support. The two materials were designed with a similar Fe load, while Ni content was 4.3 and 12% wt, respectively. OC particles in a particle size cut of 0.6–1 mm were produced via granulation and produced in sufficient quantity to be tested under relevant conditions for the scaling-up of glycerol CLR-PB reactors. XRD analysis revealed that the crystalline phases in the OC in oxidized form were FeAl_2_O_4_ and NiAl_2_O_4_ (and NiO, in the material with 12% wt Ni). The materials were completely reduced in a H_2_/N_2_ stream, forming a Ni–Fe alloy at 900 °C, and although the OC reduction reaction rate presented a high dependence on temperature, it presented sufficient reactivity and stable OTC to sustain the CLR process. They were tested in CLR of glycerol in a PB, reaching a relevant scaling-up for the technology. The materials showed high chemical stability along 100 h of operation under oxidation/reduction cycles, while complete cycles of glycerol reforming/oxidation/reduction were successfully achieved for more than 50 h of operation. Materials presented high catalytic activity in a wide range of reforming temperatures (650–850 °C), being able to achieve over 95% glycerol molar conversion to gas in very short gas/solid contact times (0.35 to 0.41 s) and up to 3 g_gly_/h g_cat_. The product gas composition approached thermodynamic equilibrium with molar ratios of H_2_/CO ranging from 5.8 at 650 °C to 3.8 at 800 °C, and no side products were detected, except for the material with 4.3% wt Ni that produced CH_4_ slightly over equilibrium at 700 °C (0.71% vol d.b.). OC stability on consecutive redox/reforming cycles was assessed on a TRL3 PB reactor at 1 and 5 bar_g._ Very consistent results were obtained in terms of product gas composition during reforming, with glycerol conversion to gas (*X*_gly_) in the range 0.96–0.99 at atmospheric pressure, and from 0.95 to 0.97 at 5 bar_g_, with low coke deposition (below 1 and 2% at 1 and 5 bar, respectively) and negligible CH_4_ formation. The results indicate that by adjusting the Ni and Fe loads in the OC/catalysts, promising materials with low Ni content can be designed and produced to sustain the energy balance of the CLR-PB of glycerol.
